# Removal of a Dental Implant Displaced into the Maxillary Sinus by Means of the Bone Lid Technique

**DOI:** 10.1155/2013/260707

**Published:** 2013-05-16

**Authors:** Pietro Fusari, Matteo Doto, Matteo Chiapasco

**Affiliations:** Unit of Oral Surgery, Department of Health Sciences, San Paolo Hospital, University of Milan, Via Beldiletto 1/3, 20142 Milan, Italy

## Abstract

*Background*. Rehabilitation of edentulous jaws with implant-supported prosthesis has become a common practice among oral surgeons in the last three decades. This therapy presents a very low incidence of complications. One of them is the displacement of dental implants into the maxillary sinus. Dental implants, such as any other foreign body into the maxillary sinus, should be removed in order to prevent sinusitis. *Methods*. In this paper, we report a case of dental implant migrated in the maxillary sinus and removed by means of the bone lid technique. *Results and Conclusion*. The migration of dental implants into the maxillary sinus is rarely reported. Migrated implants should be considered for removal in order to prevent possible sinusal diseases. The implant has been removed without any complications, confirming the bone lid technique to be safe and reliable.

## 1. Introduction

Rehabilitation of edentulous jaws with implant-supported prosthesis has become a common practice among oral surgeons and dentists in the last three decades [1].

The resorption of the alveolar ridges in the posterior maxilla and/or the maxillary sinus pneumatization often limits the available bone for positioning dental implants. To overcome these problems, the use of short implants or maxillary sinus floor lifting in association with dental implants is well documented and proved as successful procedures [2–5].

Implant displacement/migration in the paranasal sinuses, resulting from wrong planning or surgical inexperience, have been reported sporadically in the literature [6–11]. 

Implant migration into the sinuses may be followed by no relevant signs and symptoms of infection, but it can be associated with oroantral communication and/or infection that may involve the maxillary sinus and the ethmoidal, frontal, and sphenoid sinuses. These displaced foreign bodies should be removed as soon as possible to prevent such complications [12]. 

The major complication due to a foreign body in the maxillary sinus reported in the literature is sinusitis, that may bring more serious conditions such as pansinusitis, panophthalmitis, and orbital cellulitis [11, 13, 14].

Two main treatment modalities have been proposed for the removal of displaced implants in the sinuses and to treat the associated infectious complications: an intraoral approach with the creation of a window in the anterior-lateral wall of the maxillary sinus and a transnasal approach with functional endoscopic sinus surgery (FESS) [7–12].

## 2. Case Report

A 47-year-old man was referred to our department for treatment of a displaced dental implant, installed by an oral surgeon in a private dental office 30 days before, and migrated immediately after surgery into the maxillary sinus. 

**Figure 1:**
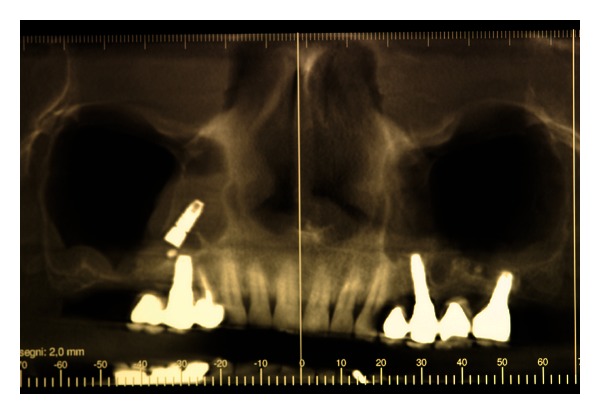
CBCT scans showing a dental implant displaced into the maxillary sinus.

**Figure 2:**
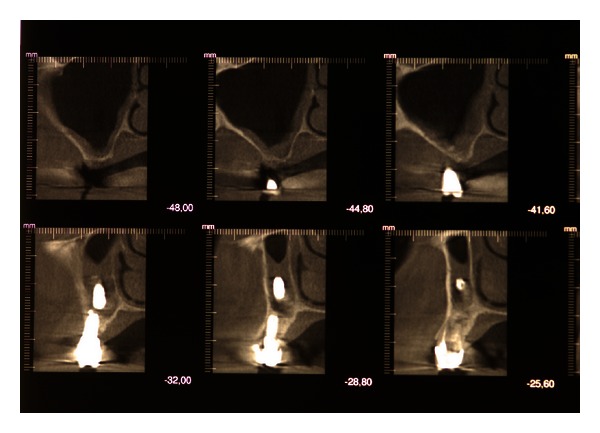
CBCT scans showing a dental implant displaced into the maxillary sinus.

The CBCT scans showed a dental implant displaced in the maxillary sinus roof, with no evidence of sinusitis (Figures 1-2). 

### 2.1. Surgical Procedure

An intraoral approach consisting im the elevation of a mucoperiosteal flap and the creation of a bony window pedicled to the Schneiderian membrane was adopted.

The patient was operated under local anesthesia. An oral antibiotic prophylaxis (amoxicillin + clavulanate, 2 g) was administered one hour prior to the start of the procedure.

**Figure 3:**
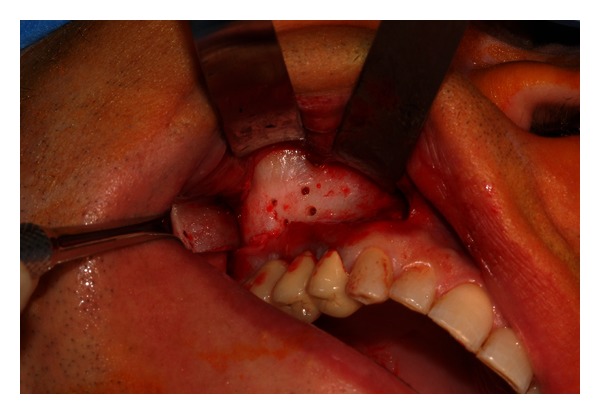
Four holes are performed after the elevation of a full-thickness flap.

**Figure 4:**
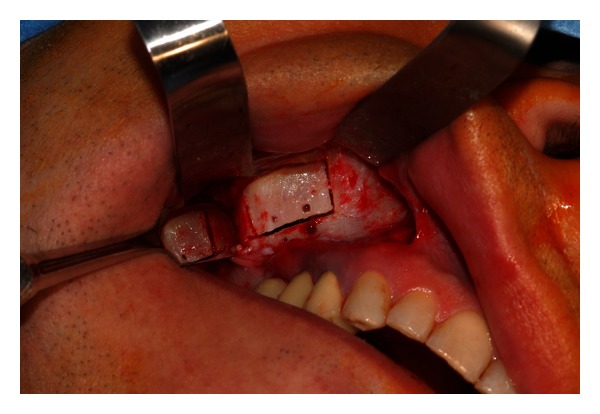
The osteotomy is performed by means of a piezoelectric instrument.

**Figure 5:**
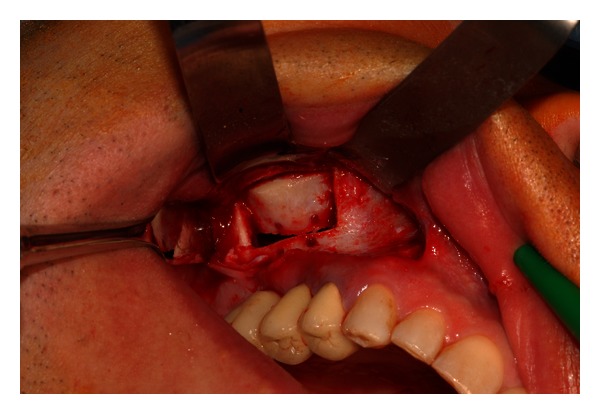
The bony window is left pedicled to the Schneiderian membrane.

The surgical intervention began with the elevation of a trapezoidal full-thickness mucoperiosteal flap. The buccal aspect of the flap was then retracted with the aid of  Langenbeck's retractor to improve the access and visibility of the maxillary sinus bony wall. A traditional rotary instrument (low-speed straight handpiece and fissure bur) was used to drill the maxillary bone with four holes (Figure 3). At this point, a rectangular osteotomy was performed using piezoelectric instruments (Figure 4). The integrity of the mucosa was maintained only along the superior side of the lid to create a pedicled window as described by Biglioli and Goisis [15] (Figure 5). 

**Figure 6:**
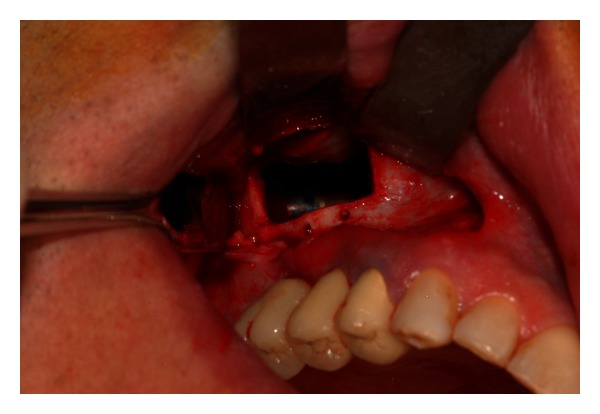
The implant is perfectly visible lying on the sinus floor.

**Figure 7:**
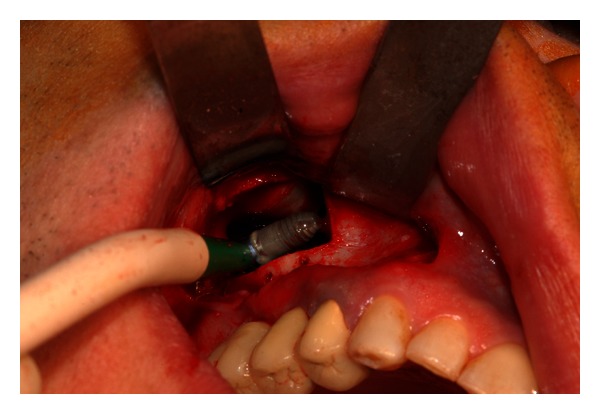
The implant is removed with a surgical aspirator.

**Figure 8:**
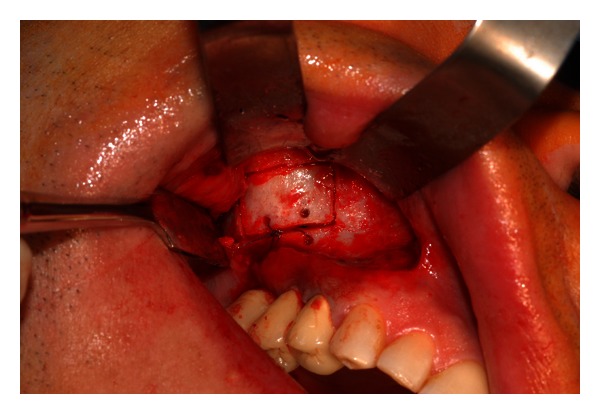
The bony segment is repositioned and secured with absorbable sutures.

The bone lid was then rotated upward; the implant was identified and removed with a surgical aspirator (Figures 6-7). The bony segment was repositioned and secured with an absorbable suture (Figure 8). After irrigation of the surgical field with sterile saline, the surgical flap was sutured, and compression with a sterile gauze was applied for a few minutes. To reduce postoperative swelling, dexamethasone (8 mg) was administered perioperatively via IM injection.

**Figure 9:**
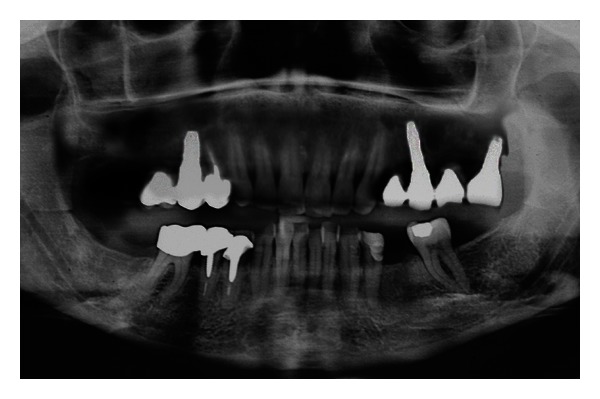
Panoramic X-ray after the intervention.

Antibiotic therapy with amoxicillin and clavulanate (1 g) was prescribed in association with nonsteroidal anti-inflammatory drugs. Chlorhexidine mouth-washes were associated to the usual oral hygiene manoeuvres for seven days. Postoperative recovery was uneventful. After seven days, the patient went through an examination and the sutures were removed. At this time, a panoramic X-ray was taken (Figure 9). 

## 3. Discussion

Surgical removal of dental implants from the maxillary sinus is not a very common oral surgery intervention. The approach proposed in this study (intraoral) is limited to the cases that do not need treatment of an obstructed maxillary sinus ostium and concomitant sinusitis of other paranasal sinuses.

Osteotomies for the bony window creation can be performed with traditional rotary instruments, or with piezoelectric instrumentation. The first method is widely used and very well documented, and it allows a fast and effective osteotomic path design. Piezoelectric instruments have been recently introduced, and they use microvibration of the surgical inserts at ultrasonic (27 to 29 kHz) frequencies to perform cutting of the hard tissues. These instruments demonstrated good cutting properties on cortical bone, allowing at the same time the preservation of soft tissues from damage in case of accidental contact [16, 17].

There are few works reported in the literature about implant migrations into the paranasal sinuses (Table 1). 

Regev et al. [6] reported three cases of implant migration, and two of them were displaced into the maxillary sinus. One occurred at the time of abutment connection due to nonosseointegration. The other was observed two months after implant placement in the anterior maxilla, where an autogenous onlay bone graft had been performed. The authors suggested that the underlying osteopenia and the occlusal forces from the maxillary denture might have contributed to the displacement in the latter case.

Iida et al. [7] reported a case of a patient who underwent dental implant installation to replace a second upper molar. Five years later, the patient noticed mobility of the implant: the prosthesis was removed from the implant, but the implant was left in position, and he underwent occlusal reconstruction of the area with an extension bridge. Eleven years later, a panoramic radiograph revealed displacement of the implant into the right maxillary sinus, and the implant was removed under local anesthesia. 

Raghoebar and Vissink [8] reported a case of a man who went through three implants installation. After three months, the migration of an implant into the maxillary sinus was discovered after a panoramic radiograph. The implant was removed in association with a sinus graft under general anesthesia.

Kitamura [9] reported a case of a woman with discomfort in the right maxilla and a discharge of pus from the nose. Panoramic radiographs and computed tomograms showed the presence of an implant in the right maxillary sinus. The patient underwent endoscopic removal of the implant under general anesthesia.

Galindo et al. [10] reported two asymptomatic cases of implant migration: one implant was kept in place after the patient refusal to undergo the operation; in the second case, the patient consented to surgical intervention, and the removal was performed 3 years later. Felisati et al. [11] reported a case of a woman who received one oral implant for the substitution of the left first upper molar, but during the surgical procedure the implant was displaced in the maxillary sinus. Owing to a delay in treatment, a spontaneous migration of the implant in the sphenoid sinus occurred. The implant was removed endoscopically through the nasal cavity.

Kluppel et al. [18] reported two cases of dental implants displaced in the maxillary sinus. One of them was removed and sinus lift performed; the other one has been kept in place with no complications after a 5-year followup.

In the literature, we can find three possible explanations of the implant migration:

1. bone resorption caused by wrong distribution of occlusal forces;
2. changes in nasal air pressure;
3. inflammatory reaction around the implant (peri-implantitis).

The majority of the authors seem to agree that the removal of a displaced implant from the sinus should be performed to avoid the possibility of development of sinus infections.

## 4. Conclusion

The migration of dental implants into the maxillary sinus is rarely reported. Migrated implants should be considered for removal in order to prevent possible sinusal diseases. The removal of displaced implants in the maxillary sinus with a buccal approach by means of a bony window creation proved to be a safe and reliable technique. 

## Figures and Tables

**Table 1:** Treatment options proposed by different authors.

Author	Implants displaced	Anatomic structures involved	Symptomatology	Treatment applied
Kluppel et al., 2010 [18]	2	Maxillary sinus	Absent	One removal, one followup
Felisati et al., 2007 [11]	1	Maxillary and sphenoid sinuses	Absent	Removal (endoscopy)
Galindo et al., 2005 [10]	2	Maxillary sinus	Absent	One removal, one followup
Kitamura, 2007 [9]	1	Maxillary sinus	Present	Removal (endoscopy)
Raghoebar and Vissink, 2003 [8]	1	Maxillary sinus	Absent	Removal + bone graft
Iida et al., 2000 [7]	1	Maxillary sinus	Absent	Removal
Regev et al., 1995 [6]	3	Maxillary sinus	Absent	Removal
